# Scattering Analysis of AlGaN/AlN/GaN Heterostructures with Fe-Doped GaN Buffer

**DOI:** 10.3390/ma15248945

**Published:** 2022-12-14

**Authors:** Dmitri S. Arteev, Alexei V. Sakharov, Wsevolod V. Lundin, Evgenii E. Zavarin, Andrey E. Nikolaev, Andrey F. Tsatsulnikov, Viktor M. Ustinov

**Affiliations:** 1 Institute of Electronics and Telecommunications, Peter the Great St. Petersburg Polytechnic University, 29 Politekhnicheskaya, 195251 Saint-Petersburg, Russia; 2 Ioffe Insitute, 26 Politekhnicheskaya, 194021 Saint-Petersburg, Russia; 3 Submicron Heterostructures for Microelectronics, Research and Engineering Center, RAS, 26 Politekhnicheskaya, 194021 Saint-Petersburg, Russia

**Keywords:** GaN, 2DEG, mobility, scattering, Fe doping

## Abstract

The results of the study of the influence of Fe segregation into the unintentionally doped GaN channel layer in AlGaN/AlN/GaN heterostructures with Fe-doped GaN buffer layer on the electrical properties of two-dimensional electron gas are presented. A set of several samples was grown by metal-organic vapor-phase epitaxy and characterized by the van der Pauw method. The dependence of concentration and mobility of the two-dimensional electron gas on the channel layer thickness was analyzed theoretically by self-consistent solving of 1D Poisson and Schrödinger equations and scattering rate calculations within the momentum relaxation time approximation. It was found that both concentration and mobility decreases were responsible for the increase in the sheet resistance in the structures with a thinner channel layer, with a drop in mobility being not only due to ionized impurity scattering, but also due to a combined effect of weakening of screening, lower carrier energy and change in form-factors on scattering by interface roughness, dislocations and polar optical phonons.

## 1. Introduction

Gallium-nitride-based high-electron-mobility transistors (HEMTs) with two-dimensional electron gas (2DEG) are very promising for high-temperature, high-frequency and high-power applications. A record low room temperature (RT) sheet resistance *R_s_* = 85 Ω/sq with the 2DEG mobility *μ* = 2470 cm^2^ V^−1^ s^−1^ and the 2DEG concentration *n* = 3 × 10^13^ cm^−2^ was reported recently for AlN/GaN structure [1]. However, to achieve a complete pinch-off, low losses and high-breakdown voltage, a semi-insulating buffer layer beneath the conductive channel is required. Intentional incorporation of deep acceptor impurities, mainly carbon or iron, is one of the approaches used to obtain high-resistivity GaN layers. Although GaN:C usually has sufficient insulating properties, carbon could induce a current collapse [2] and increased dynamic *R_ON_* [3]; the formation of pinholes is also observed [4], which could lead to low breakdown voltages.

As a dopant, Fe lacks such disadvantages, but it has other drawbacks. The main one is the Fe segregation effect, i.e., the incorporation of Fe atoms into subsequently grown layers after the Fe precursor is turned off. Unlike the magnesium memory effect, which is due to contamination of the reactor after the growth of Mg-doped layers and which can be effectively suppressed by in-situ etching [5], the source of incorporating Fe atoms is the Fe-doped sample itself [6]. As a result, a long exponential Fe tail is present in the nominally undoped layers. On the one hand, one could desire Fe to be closer to the channel to obtain higher breakdown voltages, but on the other hand, high Fe concentration in the channel region causes the 2DEG characteristics to deteriorate [7,8,9]. In that way, GaN-based HEMTs with GaN:Fe buffer is a compromise.

In this paper, we present the results of a comprehensive scattering analysis of the influence of the Fe segregation effect on the sheet resistance, 2DEG mobility and concentration of AlGaN/AlN/GaN heterostructures.

## 2. Experimental

A set of samples was grown on 2″ *c*-plane sapphire substrates by metal-organic vapor-phase epitaxy using our original in-house Dragon-125 epitaxial system with an inductively heated horizontal reactor [10,11]. The structures consisted of a low-temperature nucleation layer, a GaN:Fe buffer and an unintentionally doped GaN layer (hereafter referred to as channel layer), 1 nm AlN interlayer and 25 nm Al_0.25_Ga_0.75_N barrier. The total thickness of GaN layers was 3.4 µm. Trimethylgallium, trimethylaluminum and ammonia were used as precursors. Hydrogen, nitrogen and their mixture served as carrier gases. Ferrocene was used as a precursor to grow an Fe-doped GaN buffer layer. As we reported in our previous study [4], GaN:Fe layers remain smooth on all scales for Fe concentrations up to 1.5 × 10^18^ cm^−3^ in our growth regimes; therefore, this value was chosen in this study. The other layers were not intentionally doped. The samples were grown under the same growth conditions; the only difference between the samples was when the ferrocene flow was turned off. The sheet resistance *R_S_*, mobility μ_2DEG_ and concentration n_2DEG_ of the 2DEG were obtained by Hall effect measurements using the van der Pauw method at 77 K and RT.

## 3. Model

**Figure 1:**
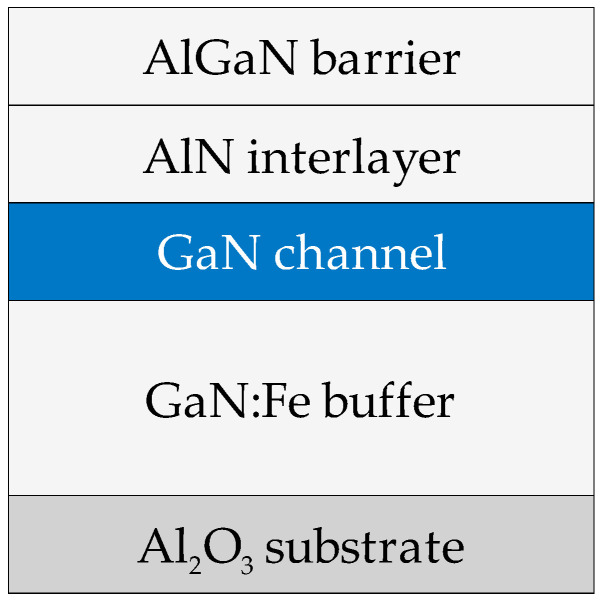
Epitaxial layer schematic.

The schematic of the epitaxial layer structure is shown in Figure 1. The 2DEG concentration, sub-band energies and wavefunctions were obtained by self-consistent solving the coupled 1D Poisson and Schrödinger equations using a predictor-corrector approach [12]. Spontaneous and piezoelectric polarization, as well as position-dependent electron effective mass were taken into account. In solving these equations, the heterointerfaces were assumed to be abrupt. The estimated interdiffusion length for the growth time and temperature used for the Al-Ga interdiffusion coefficient reported in [13] is ~0.1 nm, so the influence on the properties of the 2DEG is negligible [12], and this assumption seems reasonable. The Fe acceptor concentration was defined piecewise by a constant value throughout the buffer with exponentially decaying tail in the channel layer. The rate of decay was assumed to be 0.4 µm per decade, as was determined by secondary ion mass spectrometry (SIMS). This value agrees well with the values reported by other research groups [2,9]; however, Fe profiles could demonstrate both much smaller (<0.3 μm/dec [6]) and much larger (>0.55 μm/dec [14]) values of the rate of decay, which probably depends on growth conditions or strain. The residual donor concentration was not considered.

The low-field 2DEG mobility was calculated within the momentum relaxation time approximation as described in [15,16] and references therein. All the relevant intra- and inter-sub-band scattering mechanisms were taken into consideration, namely scattering by acoustic phonons via the deformation potential (ADP) and piezoelectric (PE) couplings, scattering by polar optical phonons (POP), ionized impurity (IMP) scattering, interface roughness scattering (IFR) and dislocation scattering (DIS). There is no evidence for the presence of Fe or Fe-nitride nanoscale clusters in GaN:Fe with Fe concentrations of 1% and lower [17]; therefore, we treated IMP scattering as scattering by single uncorrelated impurities. Alloy disorder scattering was neglected, since a 1 nm AlN interlayer almost completely eliminates the penetration of the wavefunction into the ternary AlGaN barrier [18]. In contrast to, e.g., boron-containing III-Vs [19,20] or indium-containing nitrides [21], AlGaN does not tend to phase separation/clustering; therefore, alloy cluster scattering [22] was not taken into account as well. IFR scattering was calculated using exponential autocovariance function [23]. Another mechanism that we deliberately neglected is spin-disorder scattering [24,25]. On some level, Fe-doped GaN buffer can be considered as a very dilute magnetic semiconductor. However, given a very small Fe mole fraction and a typical value of s-d exchange constant *N*_0_*α* of ~0.2 eV [26], the scattering rate is expected to be negligible.

The screening effect was taken into account for all intra-sub-band scattering mechanisms using a static multi-sub-band screening model [27,28], including POP scattering, as suggested in [27]. Moreover, it was reported that static screening gave almost the same value of POP scattering limited mobility as much more complicated dynamic screening model for the 2DEG concentrations of ~10^13^ cm^−2^ [29]. Inter-sub-band scattering was treated as unscreened. The total scattering rate for the *i*-th sub-band was calculated using Matthiessen’s rule, and the total 2DEG mobility was found as a sum over total sub-band mobilities weighted by the fractional occupancy of the sub-bands. For a proper comparison with the experimentally measured data, the Hall scattering factor for each scattering mechanism and sub-band was calculated as well [16]. For consistency, the necessary material parameters for both Poisson–Schrödinger and scattering rate calculations were taken from [30].

## 4. Results

**Figure 2:**
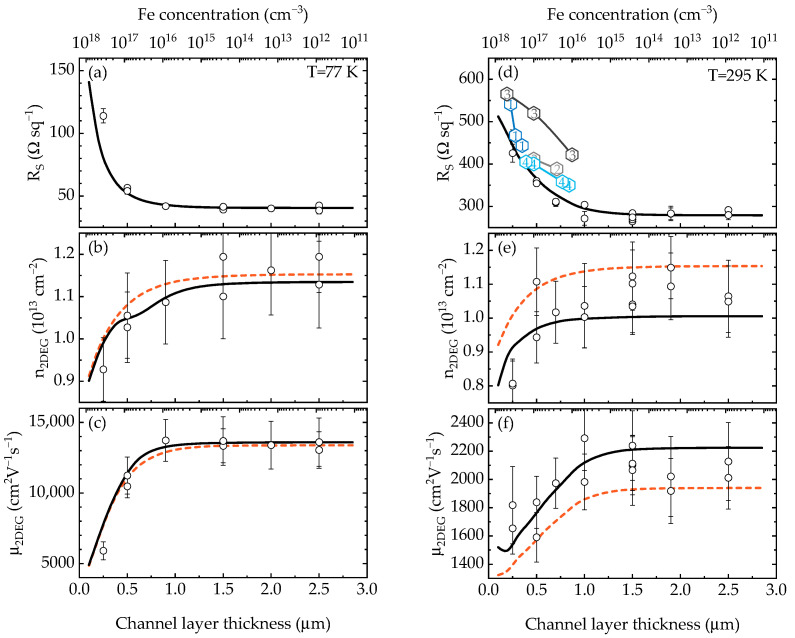
The sheet resistance, 2DEG concentration and mobility versus the GaN channel thickness at 77 K and RT ((**a**–**f**), respectively). The top X axis shows the Fe impurity concentration at AlN/GaN interface. The solid lines in (**b**,**c**,**e**,**f**) correspond to the calculated Hall concentration and mobility, while the dashed lines correspond to the calculated drift concentration and mobility. Open circles are the experimental data from this work. Hexagons with numbers in (**d**) show *R_S_* versus Fe concentration from the references: 1—[8]; 2, 3—[9]; 4—[31].

The measured sheet resistance at 77 K and RT versus GaN channel layer thickness is shown as open circles in Figure 2a,d. There is a 1.5-fold increase in *R_S_* for the structure with a 0.25 µm channel layer compared to the ones with channel layer thickness 2.5 µm at RT and an almost threefold increase at 77 K. As one can see from the rest of Figure 2, both mobility μ_2DEG_ and concentration n_2DEG_ decreased as the channel layer thickness decreased, resulting in increased *R_S_*. For comparison purpose, the data on *R_S_* versus Fe concentration at the barrier/channel interface of the structures with similar design from several references [8,9,31] are shown in Figure 2d. The trend is clearly visible, although our structures have lower *R_S_*, most likely due to the presence of AlN interlayer. In order to explain the observed results, numerical calculations were carried out.

**Figure 3:**
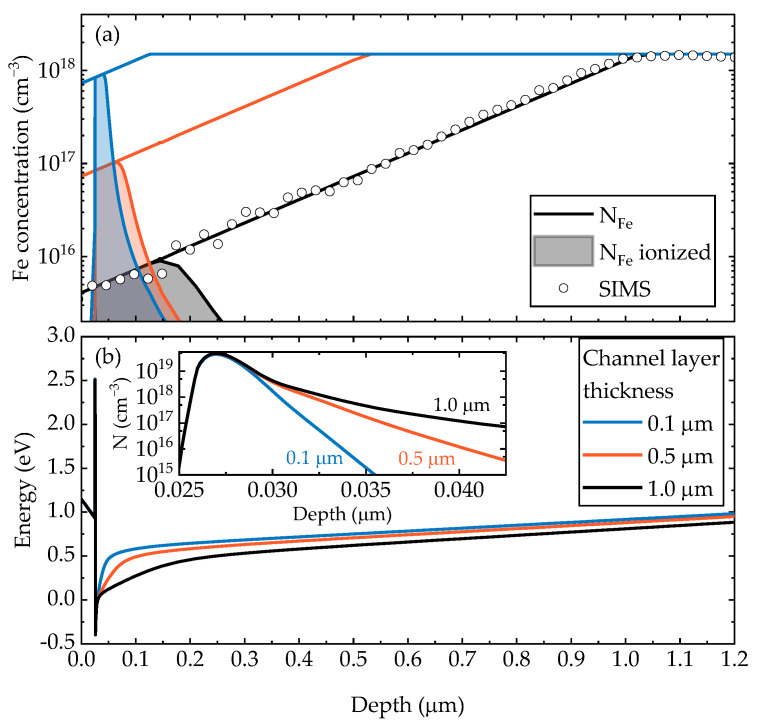
The concentration profiles of total and ionized Fe impurities (**a**) and conduction band energies (**b**) for the structures with 0.1, 0.5 and 1.0 μm channel layer. Symbols in (**a**) denote Fe impurity profile measured by SIMS. The inset shows the calculated electron concentrations in the corresponding structures.

**Figure 4:**
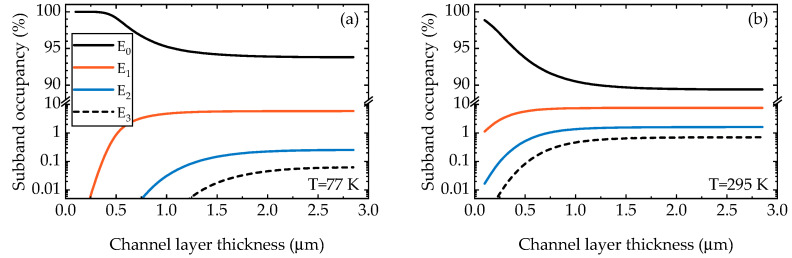
The calculated sub-band occupancies of the four lowest sub-bands at 77 K (**a**) and 295 K (**b**).

The calculated conduction band diagrams, total and ionized Fe profiles for the structures with channel layer thickness of 0.1, 0.5 and 1.0 µm are shown in Figure 3. The calculated 2DEG concentration n_2DEG_ versus the channel layer thickness at 77 K and 295 K (assumed to be RT) is shown in Figure 2b,e, respectively. The decrease in 2DEG concentration for small channel layer thicknesses is associated with a large amount of acceptor Fe impurities near the interface due to the exponential tail (Figure 3). A small discrepancy between the experimental and calculated concentration for the structure with the 0.25 μm channel layer may result from a slightly higher Fe concentration in the 2DEG region of this structure due a possible dependence of the slope of Fe tail on the total thickness of an Fe-doped buffer layer [8]. However, this is the only paper so far that reported this phenomenon, so the discrepancy may be due to random variation. As the channel layer thickness increased, the electrons began to occupy higher sub-bands, and the electron concentration profile changed (the inset in Figure 3). The sub-band occupancies at 77 K and 295 K, calculated as *n_i_/n_2DEG_* for each *i*-th sub-band, are shown in Figure 4. As can be seen, more than 99% of electrons occupy the three lowest sub-bands for any channel layer thickness even at 295 K. Therefore, we limited our mobility calculations to the three lowest sub-bands (with inter-sub-band scattering of the four lowest sub-bands taken into account).

The calculated total drift and Hall mobilities are shown in Figure 2c,f. A typical value *N_disl_* = 10^9^ cm^−2^ of dislocation density for GaN grown on sapphire substrates was assumed. The dislocation filling factor in the 2DEG region is expected to be close to 1, similarly to the bulk GaN with high free electron concentration [32]. The best fit was obtained for the root-mean-square roughness and the correlation length Δ*_RMS_* = 0.56 nm and *L* = 200 nm, respectively. These values are close to those we usually observe for the 10 × 10 μm atomic force microscopy images. However, it is disputable if RMS roughness and correlation length of the surface are directly related to those of the interface. Moreover, the measured RMS surface roughness depends on the scan area [33] due to the fractal nature of the GaN surface [34,35]. Therefore, the values of RMS and correlation length used in the paper should be considered as fitting parameters only. The corresponding calculated *R_S_* at 77 K and 295 K are shown as lines in Figure 2a,d. As one can see, a good agreement with experimental data was achieved. It can be noted that, similarly to the experimental data, there is a rather steep decrease in mobility for small channel layer thicknesses at low temperature, while the decrease at 295 K is gentler.

**Figure 5:**
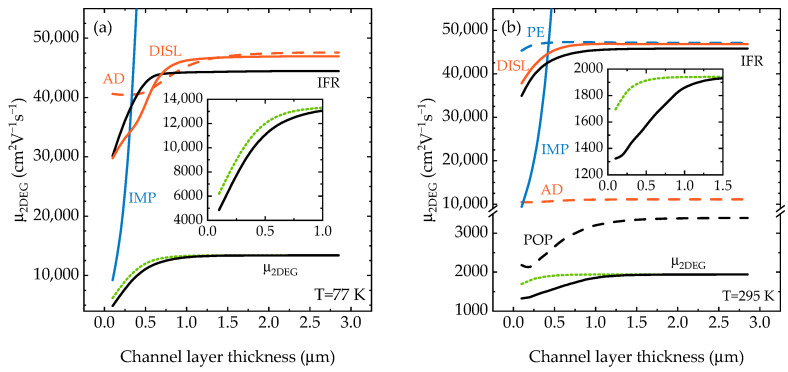
The calculated 2DEG mobilities limited by different scattering mechanisms versus the GaN channel layer thickness at 77 K (**a**) and 295 K (**b**). The green dotted lines show the total mobility, calculated taking into account the differences in IMP scattering only (see text). The insets show μ_2DEG_ vs. channel layer thickness in more detail.

The mobilities limited by different scattering mechanisms at 77 K and 295 K are shown in Figure 5. There is little wonder that ionized impurity scattering plays a significant role in structures with a very thin channel layer, since the concentration of ionized Fe impurities near the interface could be of the order ~0.5–1.0 × 10^18^ cm^−3^. However, the IMP scattering rate exponentially decreases with increased channel layer thickness. Interestingly, other scattering rates, mainly IFR, DISL and POP (at 295 K), are also higher for the structures with thinner channel layers. This is due to the combined effect of weakening of the screening, lower carrier energy and change in form-factors. IFR scattering rate is proportional to the square of the effective electric field [23], which is higher for the structures with thinner channel layer (see band diagrams in Figure 3b). In order to estimate whether the role of this effect is significant, we performed additional calculations as follows. First, we solved the Poisson/Schrödinger equation system for the structure with the thickest channel layer of 2.85 μm to obtain the wavefunction and 2DEG concentration. Then, we calculated IMP scattering limited mobility *μ_IMP_* for different channel layer thicknesses using these wavefunction and concentration. In other words, we changed only the impurity distribution in the matrix element of the Coulomb scattering, without changing the corresponding form factors, screening, carrier energies, etc. The total mobility was calculated using Matthiessen’s rule as *μ_2DEG_*^−1^ = *μ_IMP_*^−1^ + *μ*_0_^−1^, where *μ*_0_ is the total 2DEG mobility for the structure with 2.85 μm channel layer thickness from the previous calculations, and is shown in Figure 5 and the insets as green dotted lines. The obtained values are overestimated by up to 30% compared to the values obtained by the full calculations. The largest overestimation, predictably, is in the structure with the thinnest channel layer. Therefore, such simplified calculations can be useful for relatively rough estimations, while a more in-depth analysis requires full self-consistent calculations.

Band- and polarization-engineering as well as different Fe-stopping layers [9,31] may be suggested as a means to increase mobility. For example, if we assume the rate of decay of 0.04 μm instead of 0.4 μm, the calculated *R_S_* is 328 Ω sq^−1^ for the channel layer thickness of 0.1 μm. However, non-optimal regimes of the stopping layer growth could lead to high concentrations of unintentional C and Si impurities [9], possibly negating the advantage of lower Fe concentration; therefore, careful optimization is needed. Reducing the dislocation density and growing of a smoother AlN/GaN interface would definitely be beneficial as well.

## 5. Conclusions

In summary, the influence of the Fe segregation into the unintentionally doped GaN channel layer in AlGaN/AlN/GaN heterostructures with Fe-doped GaN buffer on the electrical properties of the 2DEG was investigated experimentally and theoretically. It was shown by means numerical calculations that both concentration and mobility decreases were responsible for the increase in the sheet resistance for the structures with a thinner channel layer, with the drop in mobility being not only due to ionized impurity scattering, but also due to a combined effect of weakening of the screening, lower carrier energy and change in form-factors on scattering by interface roughness, dislocations and polar optical phonons. Therefore, the use of different Fe-stopping layers, band- and polarization engineering may be suggested to further improve the characteristics of the 2DEG and GaN:Fe buffer-based HEMTs.

## Data Availability

Not applicable.
